# Dietary diversity and its determinants among women of reproductive age residing in the urban area of Nouakchott, Mauritania

**DOI:** 10.1186/s12889-024-18211-8

**Published:** 2024-03-28

**Authors:** Mariem Youssouf Issa, Yacouba Diagana, EL Kari Khalid, Sidi Mohamed Coulibaly, Alioune Gueye, Rabab. M.H. Dehah, Ould EL Kebir Mohamed Vall

**Affiliations:** 1 Research Unit of Food, Nutrition, and Metabolic Disorders, Faculty of Sciences and Technics, University of Nouakchott, Nouakchott, Mauritania; 2 Marine Ecology, Environment Nutrition and Health, Faculty of Sciences and Technics, University of Nouakchott, Nouakchott, Mauritania; 3 Joint Research Unit in Nutrition and Food, RDC-Nutrition Ibn Tofaïl University- CNESTEN, Rabat, Morocco; 4 Department of Demographic and Social Statistics, National Agency for Statistics and Demographic and Economic Analysis, Nouakchott, Mauritania

**Keywords:** Dietary diversity, Diet, MDD-W, Women of reproductive age, Mauritania

## Abstract

**Background:**

The intake of nutrient-rich foods from diverse diets ensures adequate nutrition for women. This study aims to determine dietary diversity among women of reproductive age (WRA) using the MDD-W indicator and how it relates to their socio-economic characteristics in the city of Nouakchott, Mauritania.

**Methods:**

A community-based cross-sectional study was conducted on 240 women of reproductive age, aged 15–49 years. Food consumption data were obtained through unquantified 24 h recall which is designed to identify all foods consumed by the women during this period. We computed MDD-W as the consumption of at least five out of ten predefined food groups according to the guideline of the Food and Agriculture Organization (FAO) of the United Nations. In order to determine which factors had a statistically significant influence on dietary diversity among women, we used a value of *P* < 0.05.

**Results:**

The mean of dietary diversity was 5.48 and 71.7% of WRA had an acceptable minimum dietary diversity. During the study period, 96.25% and 80% of women consumed vitamin A and iron-rich foods respectively. The consumption rate of starchy foods, vitamin A-rich fruits and vegetables, meat, fish and chicken, milk and dairy products, dark green leafy vegetables and finally other vegetables was higher; 99.6%, 75.3%, 80%, 62.5%, 60.4% and 83.3% respectively. On the other hand, the consumption of legumes, eggs and other fruits was low; at 21.7%, 14.2% and 13.8% respectively.

**Conclusions:**

The study showed that more than half of the studied population had an acceptable minimum dietary diversity. The diet was mainly based on the consumption of starchy foods, meat and other vegetables than those rich in vitamin A.

## Introduction

Maternal micronutrient deficiencies constitute a major nutritional challenge for women, affecting their health and survival as well as that of their children, particularly through intrauterine growth retardation [1, 2]. In resource poor environments, this situation is accentuated by the consumption of low quality monotonous diets.

A study by Torheim et al. in 2010 [3] showed that the risk for a range of micronutrient deficiencies is high, when grain or tuber based staple foods dominate and diets lack vegetables, fruits and animal source foods. This type of malnutrition is mainly linked to the poor quality of women’s diets, especially their lack of diversity. There is also evidence of a strong association between dietary diversity and nutrient adequacy in both developed and developing countries [4–6].

Women of reproductive age (WRA) (15–49 years) are at high risk of micronutrient deficiencies and are particularly vulnerable because of their immense micronutrient needs [1, 3].

The consumption of a varied and balanced diet during this critical period is essential, as woman’s current and future well-being can be affected by a lack of nutrients in terms of increased susceptibility to disease, reduced growth, development and productivity. In fact, micronutrient deficiencies can have a negative influence on fertility, pregnancy outcomes and congenital disabilities, compromising the health of both mother and child [7].

So far, there has been little programmatic action, despite many calls in the past decades for attention to women’s diet quality, with a specific focus on micronutrient adequacy. Among other reasons for that were, the scarcity of data on women’s dietary patterns and micronutrient deficiencies, and the lack of valid indicators to assess situations in low-income countries. The minimum dietary diversity for women (MDD-W) of reproductive age was developed by the United Nations Organization for Food and Agriculture (FAO) in 2016, as a proxy indicator to reflect dietary quality, micronutrient adequacy, showing associations with nutrient adequacy for all women of reproductive age regardless of physiological status [8–11]. In addition a manual was released to help practitioners collect data in a standardized manner definingthe MDD-W as a dichotomous indicator of whether or not women 15–49 years of age have consumed at least five out of ten defined food groups the previous day or night. According to this methodology, women who achieve minimum dietary diversity, i.e., who consume at least five food groups, are more likely to meet micronutrient intake recommendations than those who consume fewer food groups [12]. The MDD-W has been widely used to compare the dietary diversity of female populations in different contexts [9, 13, 14]. However, due to its relative short existence there are few data on the estimates of MDD-W in different contexts, and on how the indicator relates to other socioeconomic dimensions, which could inform policy makers on the relevant sectors to be targeted for achieving better women’s dietary diversity. Furthermore, while there is often a focus on food and nutrition security in rural areas, where the situation is usually more difficult, the rapid urbanization along with the global economic crisis has put many poor urban people at high risk of poor quality diets and malnutrition in developing countries [15]. Monitoring dietary diversity and quality could guide nutritional interventions that help ensure nutritional security and sustainable food production in our country, but information on micronutrient deficiencies and women’s eating habits is very scarce in Mauritania. In this context, our study aims to determine dietary diversity among women of reproductive age using the MDD-W indicator and how it relates to their socio-economic characteristics in the city of Nouakchott, Mauritania.

## Materials and methods

### Study design and settings

This was a cross-sectional survey conducted in March-April, 2022 among Mauritanian women of childbearing age (those aged between 15 and 49) [16]. The study took place in the city of Nouakchott, which is composed of three distinct wilayas (regions): Nouakchott West, Nouakchott South and Nouakchott North. Women of childbearing age, make up 26% of the total population of all these regions combined [17].

### Sample size

The sample size was calculated by the SCHWARTZ formula for cross-sectional study design [n = Z² × p (1 − p)/d2]. With p the prevalence of low dietary diversity (*p* = 7.6%) [18], Z² the accepted risk of error (1.96²) and d the desired precision (3.5%). Thus, using the proportions mentioned above, the minimum sample size of women of reproductive age was 220.

We used the prevalence of dietary diversity among women from the central Tunisian to calculate the sample size, and that was due to the lack of data related to women’s diet in Mauritania. Additionally, Tunisia is a country that is geographically close and whose population has a high degree of social similarity with the Mauritanian population.

To obtain a representative sample, a two-stage random survey was carried out. The first stage involved the random selection of households using the itinerary or random walk method [19]. The second stage consisted the random selection of one woman of reproductive age in each household from among the various members of the same category. If a woman from the selected households refused to participate, or if no woman of reproductive age was available for the interview, then the nearest house to the left was interviewed.

### Data collection methods

Our study implemented a field survey using the Food and Agriculture Organization of the United Nations (FAO) standard questionnaire for dietary diversity which was adapted to the Mauritanian context, [20]. To this, we added more specific questions related to the particular socio-economic characteristics of households in Mauritania, as well as questions specific to women such as age, level of education, etc.

Data on dietary diversity were collected using a single unquantified 24-h open recall, which is a validated approach for obtaining the necessary information on dietary diversity for calculating dietary adequacy and intake [21, 22].

Participants were asked to list all the foods they had consumed either in their household or outside their household during the last day and night. Based on the open recall, the interviewer ticked all the groups mentioned by the participant in a predefined list of foods and for any group not mentioned, the interviewer asked whether a food from that group had been consumed. Each food group consumed was counted only once, regardless of the frequency of consumption.

It should be noted that the women’s dietary diversity was calculated using a single 24-hour recall. All foods consumed by the women were classified into ten distinct food groups according to the MDD-W [12]: (1) starchy staples (grains, with roots and tubers, and plantains); (2) meat, poultry, and fish; (3) dark green leafy vegetables; (4) other vitamin A-rich fruits and vegetables; (5) other vegetables; (6) other fruits; (7) pulses (beans and lentils); (8) dairy; (9) eggs; and (10) nuts and seeds.

According to the Food and Agriculture Organization of the United Nations (FAO) guide [28], plant sources of vitamin A (VGVA) include dark green leafy vegetables, vitamin A-rich vegetables and roots, vitamin A-rich fruits and red palms, while animal sources (ANIVA) include offal, eggs, milk and milk derivatives. Iron-rich foods include offal, meat and fish.

For each woman, a minimum of 0 and a maximum of 10 points can be obtained. Higher scores indicate greater diversity, as more food groups were reported as being consumed. To achieve minimum dietary diversity, respondents must eat foods from at least five of the ten food groups.

### Quality control

The interviewers were trained in the research protocol and methodology for collecting data from household residents. The interviews were conducted by interacting directly with the women involved in each household surveyed. To ensure that the respondents understood the questions, all survey instruments were translated to the local language and tested during the interviewer training period.

### Data processing and statistical analysis

The data were entered and collected using CSPro version 7.7 software. Statistical analysis was performed using IBM SPSS Statistics version 27 for Windows [23]. MDD-W was classified into two levels of consumption for women: low if MDD-W < 5 and high if MDD-W ≥ 5. These levels were cross-referenced with certain socio-economic characteristics likely to influence women’s eating habits. To do this, a descriptive analysis was used, with a confidence level of 95%. Pearson’s chi-square test and Fisher’s exact test were used to assess the significance (at the maximum 5% level) of the results obtained. The analysis focused on the different food groups consumed by the women. Each food or food group was given a score of 1 or 0, depending on whether or not it was consumed.

### Ethical aspects

The study was approved by the ethics committee of the University of Nouakchott in Mauritania. After explaining the purpose, methodology and benefits of the study, participants provided their respective informed consent and assent.

## Results

### The socioeconomic characteristics of WRA

The sample comprises 240 women aged 15 to 49 living in 3 areas of Nouakchott (nest, south and north). Women aged 20–39 years were the most represented age group counting for 72.1%. The pregnant and breastfeeding women represented 15.4% and 22.9% respetively. Concerning educational level, 50.4% had a secondary school education. Most of the women (78.3%) have their own businesses, 25.8% owned livestock and only 8.3% owned land for housing. The percentage of women who ate out-of-home food was 13.8% (Table 1).

### The dietary diversity of WRA

**Fig. 1:**
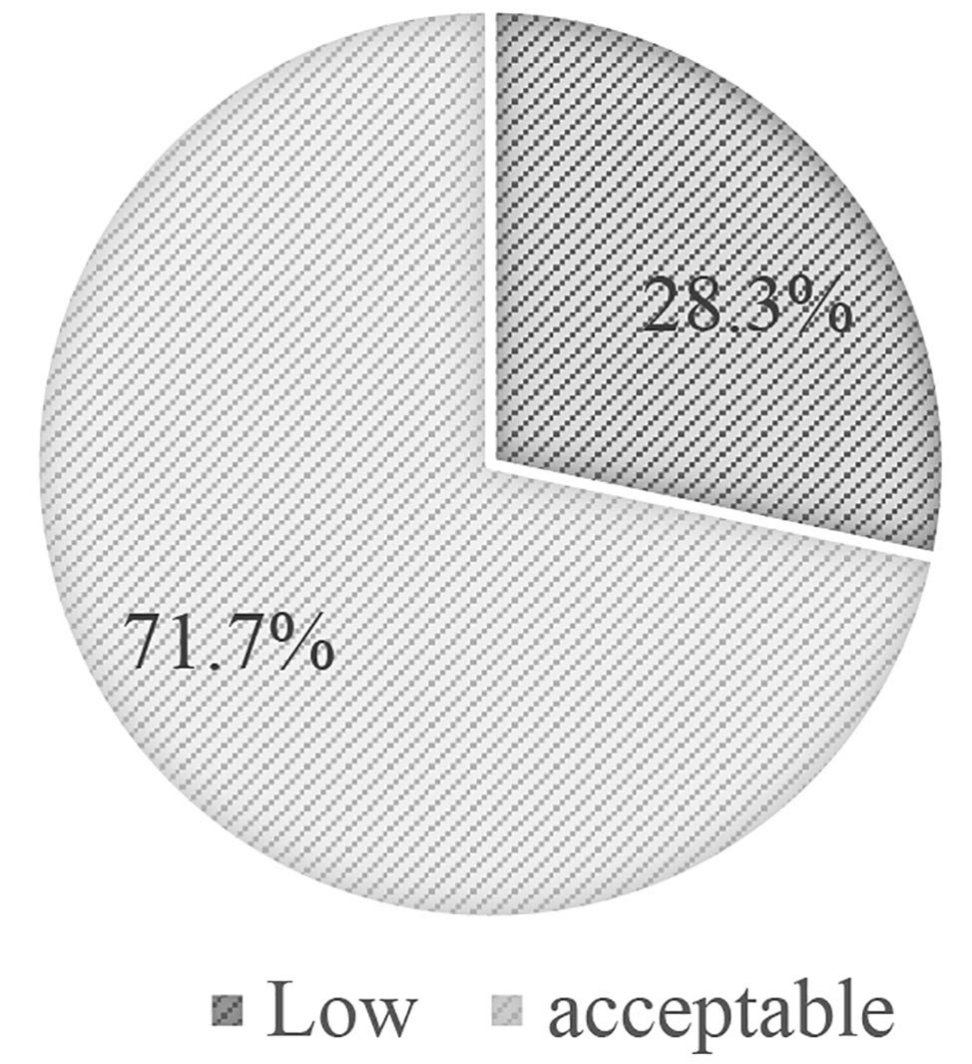
Distribution of MDD-W

The mean dietary diversity of the WRA was 5.48 (SD = 1.90 95% CI: 5.24–5.72). For pregnant and breastfeeding women, the mean was 5.89 (SD = 1.70 95% CI: 5.33–6.46) and 5.86 (SD = 1.91 95% CI: 5.32–6.40) respectively (Table 2). The distribution of different MDD-W classes revealed that 28.3% and 71.7% of the participants had low and acceptable minimum dietary diversity respectively (Fig. 1).

### The dietary diversity characteristics of WRA

**Fig. 2:**
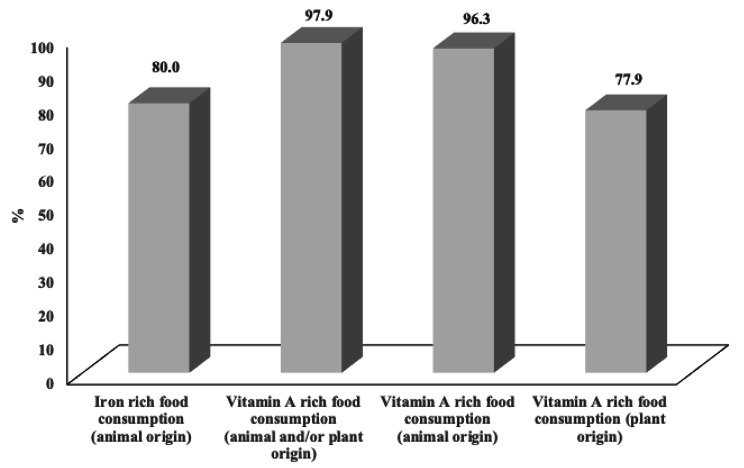
The dietary consumption of iron and vitamin A rich food

According to Table 3, the basic foods consumed daily were starchy staples (99.6%), other vegetables than those rich in vitamin A (83.3%) and meat (80.0%). The vitamin A-rich fruits and vegetables were daily consumed by 75.3%. While, 62.5% and 60.4% of women consumed dairy and dark green leafy vegetables, respectively. However, the least consumed food groups were nuts and seeds (36.3%), legumes (21.7%), eggs (14.2%) and other fruits (13.8%) (Table 3). Moreover, the consumption of food rich in iron and vitamin A was very important. Indeed, the consumption of iron rich food, vitamin A rich food of animal origin and vitamin A rich food of plant origin was 80.0%, 96.3% and 77.9%, respectively (Fig. 2).

### Determinants of minimum dietary diversity of WRA

On the other hand, it seems that women’s activities and educational level were not significant factors in determining their MDD-W (Table 4), unlike women who ate outside their houses (*P* = 0.05) or owned land for housing (*P* = 0.046), or owned cattle (*P* = 0.009) who presented a good MDD-W compared to women who usually consumed meals in house or did not own land or cattle, respectively (Table 4).

## Discussions

This study revealed the minimum dietary diversity of WRA (MDD-W) residents in Nouakchott, Mauritania. Various socio-economic determinants of dietary diversity have also been identified. Our study findings indicated that both the mean dietary diversity (5.48) of women and the proportion of women who reached the MDD-W (71.7%) were acceptable. The women’s diet was largely dominated by starchy staples followed by other vegetables and meat, fish and poultry, while eggs, other fruits and pulses were reported by few women. Women’s out of home consumption, land for housing ownership and cattle ownership were significantly associated with their dietary diversity.

The mean dietary diversity of women was slightly higher on average than the 5-point cut-off proposed by FAO [12]. This study showed a better result than those obtained by several other studies conducted in Africa, eight Latin-American countries and St. Martin Island in Bangladesh populations using the same methodology [24–28]. When using the MDD-W threshold of five or more food groups, 71.7% of WRA living in urban Mauritania have succeeded in achieving the minimum dietary diversity and hence are more likely to have adequate micronutrient intake. This percentage exceeds 40.3%, 31% and 25% of the WRA reaching the minimum dietary dieversity in Hafizul Islam, Bangladesh [28], Custodio, Burkina Faso [24] and Chakona, South Africa [25] respectively. This variation in MDD-W between regions is probably due to differences in the study periods and geographical areas [28].

In addition, our study revealed that women’s diets were largely dominated by starchy staples, followed by other vegetables, meat, fish and poultry. These food groups are consumed at very high rates compared to other food groups, such as vitamin A-rich fruits and vegetables, dairy and dark green leafy vegetables. This pattern of consumption in our study population is consistent with descriptions found in most previous studies in Burkina Faso, South Africa… [24, 25, 28, 29]., although variations in the consumption rates of each food group are observed.

In our context, the high consumption of plant products, particularly cereals, can be explained by their availability and/or affordability, as well as by their integration into local dietary habits for centuries. This finding is in line with the results reported by some authors concerning the consumption of Ethiopian women [30, 31]. On the other hand, consumption of certain products, sometimes new to the local diet, is declining. These include fruits, nuts and seeds, and their low consumption is probably due to their high cost outside their harvesting season, as well as to other considerations such as local availability. In Nouakchott, access to these products may be limited specifically for certain families with low incomes, due to the transport costs or customs fees, especially if they are imported.

The study also revealed a positive association between out of home consumption and dietary diversity, although these results diverge from the findings of other studies that suggested less dietary diversity in individuals consuming meals out of the home [32, 33]. This difference may be attributed to the availability of more varied foods in restaurants compared to those at home for those who ate at a restaurant [34], or to food consumption habits outside the home, which may influence household dietary diversity [35]. Moreover, the study identified the residential land ownership and cattle ownership as significant factors for MDD-W determination. Women who owned land for housing and those who owned cattle showed significantly higher levels of MDD-W. This result does not exactly coincide with the findings of a study carried out in Pakistan, which suggested that dietary diversity does not necessarily vary according to economic status [36].

It is important to note that the MDD-W in our study was not associated with certain factors such as area of residence, age, physiological status, woman’s activity or level of education.

Indeed, our results indicate that the level of education is not associated with MDD-W, which differs from the findings of studies carried out in Belgium, China, Burkina Faso and South Africa [37–39], where women’s level of education has a significant influence on their knowledge, skills and practices with regard to food diversity, health and hygiene [40]. This divergence could be attributed to the overriding influence of local behaviors on eating habits. In fact, our women, particularly those from the Maurish ethnic group, are often influenced by the traditional idea that weight gain is associated with prestige and beauty, leading them to frequently adopt harmful measures to stimulate their appetite and increase their food intake, without necessarily diversifying it known their major role in food purchasing decisions [41]. This risk of failure to diversify is higher for women with less autonomy and from poor or low-income households [42, 43].

Crucially, food prohibitions, particularly among uneducated and sub-Saharan African women, have been identified as one of the factors contributing to maternal undernutrition during pregnancy [44–48].

**Table 1:** Women’s socio-economic characteristics

Variable and Categories		Frequency (n)	Percentage (%)
Age	15–19	36	15.0
	20–29	103	42.9
	30–39	70	29.2
	40–49	31	12.9
Level of education	None	23	9.6
	Koranic	14	5.8
	Primary school	63	26.3
	Secondary school	121	50.4
	High school or college	19	7.9
Area	NCK-South	110	45.8
	NCK- West	42	17.5
	NCK- North	88	36.7
Woman’s activity	Agriculture	1	0.4
	Own business	188	78.3
	Trade	26	10.8
	House maid	15	6.3
	Public employee	4	1.7
	Private employee	6	2.5
Own land for housing		20	8.3
Cattle ownership		62	25.8
Breast feeding women		50	20.8
Pregnant women		37	15.4
Out-of-home food consumption		33	13.8

NCK: Nouakchott

**Table 2:** Mean of dietary diversity

Variable	Category	Mean	SD	95% CI	Min-Max	*P*-value
All		5.48	1.90	(5.23–5.72)	[1-10]	
Women (15–49 years)	15–19	5.69	2.27	(4.93–6.46)	[2-10]	0.697
	20–29	5.49	1.92	(5.11–5.86)	[2-10]	
	30–39	5.61	1.74	(5.19–6.02)	[1-10]	
	40–49	4.90	1.68	(4.28–5.51)	[1-8]	
Breast feeding women	Yes	5.86	1.91	(5.32–6.40)	[1-10]	0.174
	No	5.38	1.89	(5.10–5.64)	[2-10]	
Pregnant women	Yes	5.89	1.70	(5.32–6.45)	[1-10]	0.053
	No	5.40	1.93	(5.13–5.67)	[2-10]	

*P*-value: chi square test. SD: standard deviation. CI: confidence interval

**Table 3:** Food groups consumed by WRA

Food type	Consumption (n)	Percentage of consumption (%)
Starchy staples	239	99.6
Vitamin A-rich fruits and vegetables	183	75.3
Dark green leafy vegetables	145	60.4
Other vegetables	200	83.3
Other fruits	33	13.8
Nuts and seed	87	36.3
Meat, fish and chicken	192	80
Eggs	34	14.2
Pulses	52	21.7
Dairy	150	62.5

WRA: Women of reproductive age

**Table 4:** Socio-economic characteristics and the MDD-W

Variable Category		(%) MDD-W Low < 5	(%)MDD-W Acceptable > = 5	*P*- value
Area	NCK-South	30.0	70.0	0.863
	NCK- West	26.2	73.8	
	NCK- North	27.3	72.7	
Breast feeding women	Yes	22.0	78.0	0.174
	No	30.0	70.0	
Pregnant women	Yes	16.2	83.8	0.053
	No	30.5	69.5	
Level of education	None	39.1	60.9	0.585
	Koranic	14.3	85.7	
	Primary school	27.0	73.0	
	Secondary school	28.1	71.9	
	High school or college	31.6	68.4	
Woman’s activity	Agriculture	0.0	100.0	0.212
	Own business	28.7	71.3	
	Trade	19.2	80.8	
	House maid	46.7	53.3	
	Public employee	25.0	75.0	
	Private employee	16.7	83.3	
Out-of-home consumption	Yes	15.2	84.8	0.05*
	No	30.4	69.6	
Land for housing ownership	Yes	5.0	95.0	0.046*
	No	30.5	69.5	
Cattle ownership	Yes	19.4	80.6	0.009*
	No	31.5	68.5	

### Study limits

This study did not consider the quantity of food consumed by women thus we can’t conclude wether food intake will be sufficient to meet the needs of those women or not even if the consumption of food frequency exceeded 80% for some micronutrient-rich foods. Another important limitation is that the study didn’t cover all seasons in Mauritania. Finally cultural beliefs may also have influenced our findings, as some women were conservative and reluctant to discuss their dietary beliefs and practices particularly during pregnancy.

## Conclusion

The prevalence of MDD-W in urban Nouakchott in Mauritania was 71.7%, which means that more than half of the studied women had a diverse diet. The diet was mainly based on the consumption of starchy foods, meat, fish and poultry, other vegetables, vitamin A rich fruits and vegetables, dairy and dark green leafy vegetables. In our study, the MDD-W was significantly related to out-of-home consumption, land and cattle ownership. On the other hand, education level, age, area of residence, and women’s activity were not significant determinants of MDD-W.

## Data Availability

The datasets used and/or analyzed during the current study available from the corresponding author on reasonable request.

## Abbreviations

MDD-W: minimum dietary diversity for women
WRA: women of reproductive age
NCK: Nouakchott
VGVA: vitamin A-rich foods of plant origin
ANIVA: vitamin A-rich foods of animal origin
VITA: Vitamin A-rich foods
FAO: Agriculture Organization of the United Nations
